# The botanical drug PBI-05204, a supercritical CO_2_ extract of *Nerium oleander*, sensitizes alveolar and embryonal rhabdomyosarcoma to radiotherapy *in vitro* and *in vivo*

**DOI:** 10.3389/fphar.2022.1071176

**Published:** 2022-12-01

**Authors:** Sara Vaccaro, Alessandra Rossetti, Antonella Porrazzo, Simona Camero, Matteo Cassandri, Silvia Pomella, Miriam Tomaciello, Giampiero Macioce, Francesca Pedini, Giovanni Barillari, Cinzia Marchese, Rossella Rota, Giovanni Cenci, Mario Tombolini, Robert A. Newman, Peiying Yang, Silvia Codenotti, Alessandro Fanzani, Francesca Megiorni, Claudio Festuccia, Giuseppe Minniti, Giovanni Luca Gravina, Francesca Vulcano, Luisa Milazzo, Francesco Marampon

**Affiliations:** ^1^ Department of Radiotherapy, Policlinico Umberto I, “Sapienza” University of Rome, Rome, Italy; ^2^ Department of Oncology and Molecular Medicine, Istituto Superiore di Sanità, Rome, Italy; ^3^ Department of Biotechnological and Applied Clinical Sciences, Laboratory of Radiobiology, University of L’Aquila, L’Aquila, Italy; ^4^ Department of Oncohematology, Bambino Gesù Children’s Hospital, IRCCS, Rome, Italy; ^5^ Department of Maternal, Infantile and Urological Sciences, “Sapienza” University of Rome, Rome, Italy; ^6^ Department of Clinical Sciences and Translational Medicine, University of Rome Tor Vergata, Rome, Italy; ^7^ Department of Experimental Medicine, “Sapienza” University of Rome, Rome, Italy; ^8^ Dipartimento Biologia e Biotecnologie “C. Darwin,” “Sapienza” University of Rome, Rome, Italy; ^9^ Istituto Pasteur Italia-Fondazione Cenci Bolognetti, Rome, Italy; ^10^ Otolaryngology Department, San Giovanni-Addolorata Hospital, Rome, Italy; ^11^ Phoenix Biotechnology Inc., San Antonio, TX, United States; ^12^ Department of Palliative, Rehabilitation and Integrative Medicine, The University of Texas MD Anderson Cancer Center, Houston, TX, United States; ^13^ Department of Molecular and Translational Medicine, Division of Biotechnology, University of Brescia, Brescia, Italy; ^14^ Radiation Oncology Unit, Department of Medicine, Surgery and Neurosciences, University of Siena, Siena, Italy; ^15^ IRCCS Neuromed, Pozzilli, IS, Italy; ^16^ Department of Biotechnological and Applied Clinical Sciences, Radiotherapy Oncology Unit, St Salvatore Hospital, University of L’Aquila, L’Aquila, Italy

**Keywords:** PBI-05204, oleandrin, rhabdomyosarcoma, Na/K +ATPase, radiotherapy, radiosenisitizing agent

## Abstract

Treatment of rhabdomyosarcoma (RMS), the most common a soft tissue sarcoma in childhood, provides intensive multimodal therapy, with radiotherapy (RT) playing a critical role for local tumor control. However, since RMS efficiently activates mechanisms of resistance to therapies, despite improvements, the prognosis remains still largely unsatisfactory, mainly in RMS expressing chimeric oncoproteins PAX3/PAX7-FOXO1, and fusion-positive (FP)-RMS. Cardiac glycosides (CGs), plant-derived steroid-like compounds with a selective inhibitory activity of the Na^+^/K^+^-ATPase pump (NKA), have shown antitumor and radio-sensitizing properties. Herein, the therapeutic properties of PBI-05204, an extract from *Nerium oleander* containing the CG oleandrin already studied in phase I and II clinical trials for cancer patients, were investigated, *in vitro* and *in vivo*, against FN- and FP-RMS cancer models. PBI-05204 induced growth arrest in a concentration dependent manner, with FP-RMS being more sensitive than FN-RMS, by differently regulating cell cycle regulators and commonly upregulating cell cycle inhibitors p21^Waf1/Cip1^ and p27^Cip1/Kip1^. Furthermore, PBI-05204 concomitantly induced cell death on both RMS types and senescence in FN-RMS. Notably, PBI-05204 counteracted *in vitro* migration and invasion abilities and suppressed the formation of spheroids enriched in CD133^+^ cancer stem cells (CSCs). PBI-05204 sensitized both cell types to RT by improving the ability of RT to induce G2 growth arrest and counteracting the RT-induced activation of both Non‐Homologous End‐Joining and homologous recombination DSBs repair pathways. Finally, the antitumor and radio-sensitizing proprieties of PBI-05204 were confirmed *in vivo*. Notably, both *in vitro* and *in vivo* evidence confirmed the higher sensitivity to PBI-05204 of FP-RMS. Thus, PBI-05204 represents a valid radio-sensitizing agent for the treatment of RMS, including the intrinsically radio-resistant FP-RMS.

## Introduction

Rhabdomyosarcoma (RMS) is the most common pediatric soft tissue sarcoma. The two major subtypes are the alveolar (ARMS), more frequently expressing the pro-oncogenic fusion proteins PAX3/7-FOXO1 (PAX3/7-FKHR), namely “fusion positive” RMS (FP-RMS), and the embryonal (ERMS), characterized by different mutations, “fusion negative” (FN-RMS). However, despite the status of fusion proteins, ARMS and ERMS present similar molecular perturbations, this indicating some commonality in the molecular driving forces in RMS (Sorensen et al., 2002; Davicioni et al., 2009; Rudzinski et al., 2015, 2017; Skapek et al., 2019). Treatment of RMS, currently consists of neoadjuvant radiotherapy (RT), with or without adjuvant chemotherapy (CHT), followed by a delayed excision (PDQ Pediatric Treatment Editorial Board, 2002; Cecchetto et al., 2007; Gallego et al., 2021). RT is critical to improve survival in RMS patients (Terezakis and Wharam, 2013; Mandeville, 2019). However, RMS has been shown to aberrantly express several mechanisms that sustain the resistance to RT (Marampon et al., 2011; 2019b; 2019c; 2019a; Ciccarelli et al., 2016; Gravina et al., 2016; Megiorni et al., 2017; Camero et al., 2019, 2020, 2021; Giannattasio et al., 2019; Petragnano et al., 2020a; 2020b; Casey et al., 2021; Cassandri et al., 2021; Codenotti et al., 2021; Rossetti et al., 2021; Perrone et al., 2022)., potentially responsible of the high relapse rate after apparent complete remission (Heske and Mascarenhas, 2021). Notably, the use of larger dose of radiations, has not improved the therapeutic efficiency of radiation (Kalbasi et al., 2020; Parsai et al., 2020) suggesting that new radiosensitizing strategies are urgently needed in order to improve patient overall survival.

RT kills cancer cells by inducing the accumulation of potentially repairable DNA single strand breaks (SSBs) and their transformation into non-repairable DNA double strand breaks (DSBs) (Baskar et al., 2014). cancer cells can efficiently repair SBSs, preventing the formation of DSBs formation, ability that result to be higher in the cancer stem cell (CSC) subpopulations (Wang, 2015), thus resulting the real responsible of intrinsic radioresistance (Rycaj and Tang, 2014; Wang, 2015; Schulz et al., 2019; Arnold et al., 2020). Cardiac glycosides (CGs) (e.g., digitoxin, digoxin, ouabain, and oleandrin) are selective inhibitors of the Na^+^/K^+^-ATPase pump (NKA), , commonly used to treat heart failure (Pavlovic, 2020). The aberrant expression/activity of NAK has been found in several cancer types (Mijatovic et al., 2012; Durlacher et al., 2015), including RMS (Liepkans, 1990). Thus, CGs have shown towards some types of malignant tumors, both *in vitro* and *in vivo* (López-Lázaro et al., 2005; Kumavath et al., 2021), working at concentrations commonly found in the plasma of cardiopathic patients treated with CGs (López-Lázaro et al., 2005). Thus, CGs have been tested in clinical trials for the treatment of cancer demonstrating satisfactory safety and efficacy (Mekhail et al., 2006; Menger et al., 2013; Hong et al., 2014; Frankel et al., 2017; Roth et al., 2020). Furthermore, considering that CGs to act as potent inhibitors of DSB repair (Wha Jun et al., 2013; Surovtseva et al., 2016; Tian et al., 2020), increasing evidence suggests their use as effective radiosensitizers (Verheye-Dua and Böhm, 1998; Nasu et al., 2002; Lee et al., 2017; Zhang et al., 2017; Du et al., 2018; Colapietro et al., 2022). Notably no studies have been still conducted on RMS.

We have recently show that PBI-05204, a defined supercritical CO_2_ extract of *N. oleander*, has anticancer and radiosensiting effects towards glioblastoma (Colapietro et al., 2020; Colapietro et al., 2022). Herein, we have investigated the therapeutic potential of PBI-05204, alone and in combination with RT, towards RMS, by using, *in vitro* and *in vivo,* RD (FN-RMS) and RH30 (FP-RMS), the most representative RMS cell lines. Herein we found that PBI-05204 efficiently counteracted the transformed and intrinsically radioresistant phenotype of RMS by concomitantly inducing cytostatic and cytotoxic effects, promoting RT-induced G2 cell cycle arrest and restraining the ability of RMS cells to repair RT-induced DNA damage. Notably, PBI-05204 showed important *in vivo* effects, enhanced by RT. Altogether, these results suggest that PBI-05204 could have therapeutic and radiosensitizing properties on RMS.

## Materials and methods

### Cell lines and pharmacological treatment

RD (ERMS, FN-RMS) and RH30 (ARMS, FP-RMS) human cell lines were purchased from American Type Culture Collection (Manassas, VA, United States), cultured, in Dulbecco’s Modified Eagle’s and RPMI medium (DMEM) containing 10% Fetal Bovine Serum (Hyclone, Logan, UT, United States) supplemented with glutamine and gentamycin (GIBCO-BRL Gaithersburg, MD, United States), dissociated using 0.25% trypsin and 0.02% EDTA solution and resuspended into a fresh medium once every 2–3 days (Camero et al., 2019 b). GenePrint 10 System (Promega Corporation, Madison, WI, United States) was used to authenticate cell cultures by comparing the DNA profiles of cell lines with those found in GenBank. Multipotent mesenchymal stromal cells (MSCs) were previously described (Vulcano et al., 2016). The supercritical CO_2_ extract of *N. oleander* PBI-05204 was provided by Phoenix Biotechnology, Inc., (San Antonio, Texas) and characterized by using an AccuTOF-DART mass spectrometer (Jeol UAS, Peabody, MA). Specific molecular content of the extract was previously reported (Siddiqui et al., 1995; Dunn et al., 2011).

### Viability of cells

RD and RH30 cells were seeded into 6-well tissue culture plates at a density of 8,500 cells/cm^2^ and treated with PBI-05204 24 (hours) h later. Trypan blue (Thermofisher) dye exclusion test was used to assess cell viability. A Countess II Automated Cell Counter (ThermoFisher Scientific, Waltham, MA, United States) was used to assess the number of the cells. “Quest Graph™ IC_50_ Calculator” (AAT Bioquest, Inc.,) was used to calculate IC_50_ values (AAT Bioquest, 2022).

### Migration and invasion assays

Migration was assessed using wound healing assays that were performed as previously described (Gravina et al., 2017). Briefly, RD and RH30 cells were plated in 6-well plates and incubated with or without PBI-05204 for 24 h. The following day, a sterile pipette tip was used to scratch the cell monolayer (4–5 parallel scratches/plate). Cells were washed with PBS, photographed to mark scratched tracks, and incubated for an additional 24 h to evaluate cell migration into the injured areas. Wound healing was quantified using ImageJ 1.47v software. For the invasion assay, RD and RH30 cells (8 × 10^5^ cells/ml) were seeded in the upper portion of a Boyden chamber separated from the lower compartment, containing DMEM with 10% FBS added with PBI-05204 (IC_50_) or DMSO, by a matrigel-coated PVP-free polycarbonate filter with 8 mm pore size (Costar, Cambridge, United States). After incubation at 37°C for 6 h, migrated cells were stained with Diff-Quik (Dade-Behring, Milan, Italy) (Codenotti et al., 2019). The number of migrated cells was quantified using ImageJ 1.47v software. Experiments were carried out in triplicate.

### Sphere culture and sphere formation

Sphere-forming cells were obtained as previously described (Ciccarelli et al., 2016; Camero et al., 2020; Megiorni et al., 2021). Briefly, RD and RH30 cells were cultured in anchorage-independent conditions (ultra-low attachment flasks or plates, Corning) in stem cell (SC)-medium consisting of DMEM:F12 medium (Gibco-Invitrogen) and B27 (ThermoFischer). Fresh human epidermal growth factor (20 ng/ml) and fibroblast growth factor (20 ng/ml) (PeproTech, London, United Kingdom) were added twice/week until cells formed floating spheres. To evaluate the primary sphere formation, cells from sub-confluent (70–80%) monolayer cultures were plated at a density of 100, 500 or 1,000 cells in a 24-well culture plate (Corning Inc., Corning, NY, United States). For the sphere formation assay, the number of primary tumor spheres was determined.

### Flow cytometer analysis of cell cycle distribution and stem cell markers

For cell cycle analysis, a BD Cycletest Plus DNA Kit (BD Biosciences) was used for DNA staining. Following trypsinization, cells were adjusted to a concentration of 1×10^6^ cells/ml and treated using reagent kit, according to the manufacturer’s instructions. The cell cycle status was analyzed by flow cytometry using propidium iodide (PI). Analysis was performed using a flow cytometer (FACSCalibur), and the cell-cycle distribution was analyzed using the Mod-Fit LT software (Verity Software House, Topsham, ME, United States). Stem cell markers in RMS cells were evaluated by staining with monoclonal antibodies conjugated with phycoerythrin (PE) anti–CD133 (BD Biosciences, Buccinasco, Italy). Appropriate isotype controls for non-specific binding were used for each antibody. A minimum of 50,000 events were acquired for each sample by the flow cytometer and the CellQuest software (BD Biosciences) was used for both data acquisition and analysis (Marampon et al., 2019d).

### Protein extraction,western blot and protein simple WES western analysis

For total protein extraction, RD and RH30 cells were lysed in 2% SDS containing 2 mM phenyl-methyl sulphonyl fluoride (PMSF) (Sigma-Aldrich (St. Louis, MO, United States), 10 μg/ml antipain, leupeptin and trypsin inhibitor, 10 mM sodium fluoride and 1 mM sodium orthovanadate (all from Sigma-Aldrich, St. Louis, MO, United States) and sonicated for 30 s (sec). Protein concentration was estimated by BCA assay and equal amounts were separated on SDS-PAGE. Proteins were transferred to a nitrocellulose membrane (ThermoFisher Scientific, Waltham, MA, United States) by electroblotting. The balance of total protein levels was confirmed by staining the membranes with Ponceau S (Sigma-Aldrich (St. Louis, MO, United States). Membranes were blocked for 1 h in 5% non-fat dry milk in Tris-buffered saline and Tween-20 (TBS-T) and then incubated at 4 °C overnight with primary antibodies. The primary antibodies used were: p21^Waf1/Cip1^ (C-19) and p27^Cip1/Kip1^ (F-8), Cyclin A (BF683), Cyclin B1 (H-20), Cyclin E (HE12), myelocytomatosis virus oncogene cellular homolog (c-Myc) (9E10), N-Myc (B.8.4.B), phosphorylated extracellular signal-regulated kinase 1/2 (ERK1/2^PO4^) (E-4), extracellular signal-regulated kinase (ERK1/2) (C-14), phosphorylated protein kinase B (Akt^PO4^) (C-11), e phosphorylated protein kinase B (Akt) (5C10), and vinculin (7F9) by Santa Cruz Biotechnology (Dallas, TX, United States). Appropriate horseradish peroxidase (HRP)-conjugated secondary antibodies (Santa Cruz Biotechnology (Dallas, TX, United States) were used for 1 h at room temperature (Gravina et al., 2019; Menna et al., 2022). Western blots for ATM, ATM^PO4^, DNA-PKCs, DNA-PKCs^PO4^ and vinculin were performed using a Protein Simple WES Western instrument (San Jose, CA). Cell and tissue lysates were prepared as described above. Protein simple (6 µl) was mixed with 5x fluorescent master mix (Protein Simple) to achieve a finial concentration of 1x master mix buffer according to manufacturer’s instructions. Samples were then denatured at 95°C for 5 min. All materials and solutions added onto the assay plate were purchased from Protein Simple except primary antibodies. Antibody diluent (10 µl), protein normalizing reagent, primary antibodies, secondary antibodies, chemiluminescent substrates, 3 µl of sample, and 500 µl of wash buffer were prepared and dispensed into the assay plate. Assay plates were loaded into the instrument and proteins were separated within individual capillaries. Protein detection and digital images were collected and analyzed with Compass software (Protein Simple) and data were reported as area under the peak, which represents the intensity of the signal. For primary antibody, phospho-ATM (D6H9, Ser 1981, used at 1:25), phospho-DNA-PKCs (E9J4G, Ser 2056, used at 1:25) and DNA-PKCs (E6U3A, used at 1:100) by Cell Signaling (Danvers, MA, United States); ATM (G-12, used at 1:50) by Santa Cruz Biothecnology (Dallas, TX, United States) were mixed with vinculin (hVIN-1, used at 1:100) by Sigma-Aldrich Inc. (Saint Louis, MO, United States). Anti-mouse HRP and anti-rabbit HRP secondary antibodies from Protein Simple were used (Kannan et al., 2018; Hui et al., 2020). Quantification of Western blot data was performed by using a ChemiDoc MP (Bio-Rad) imager.

### Radiation exposure and clonogenic assay

Radiation was delivered at room temperature using a x-6 MV photon linear accelerator. The total single dose of 4 Gy was delivered with a dose rate of 2 Gy/min using a source-to-surface distance (SSD) of 100 cm. A plate of Perspex thick 1.2 cm was positioned below the cell culture flasks in order to compensate for the build-up effect. Tumor cells were then irradiated placing the gantry angle at 180°. Non-irradiated controls were handled identically to the irradiated cells with the exception of radiation exposure. The absorbed dose was measured using a Duplex dosimeter (PTW). For clonogenic survival assay, exponentially growing RD and RH30 cells in 25-cm^2^ flasks were treated with PBI-05204 or vehicle and irradiated 24 h later. Three h after irradiation, cells were harvested, counted, diluted serially to appropriate densities, plated in triplicate in six multi-well plates with 2 ml of complete drug-free medium/each well. Fourteen days (d) later, cells were fixed with methanol:acetic acid (10:1, v/v), and stained with crystal violet. Colonies containing >50 cells were counted.

### Animal research ethics statement and in vivo xenograft experiments

The recommendations of the European Community (EC) guidelines (2010/63/UE and DL 26/2014 for the use of laboratory animals) and the Istituto Superiore di Sanità guidelines, complying with the Italian government regulation n.116 27 January 1992 for the use of laboratory animals code 221/2022-PR (D9997.140) approved 4 April 2022 were followed to undertake *in vivo* experiments. Before any invasive manipulation, mice were anesthetized with a mixture of ketamine (25 mg/ml)/xylazine (5 mg/ml). For xenotransplants exponentially growing RD or RH30 cells were detached by trypsin-EDTA, washed twice in PBS, and resuspended in saline solution at cell densities of 1 × 10^6^/200 μl. Xenotransplants were done in 45-day-old female nude CD1 mice from Charles River Laboratories Italia, SRL (Calco, Italy), by subcutaneously injection in the leg using a 21-gauge needle on a tuberculin syringe. Treatments were started when tumors reached a volume of 0.3–0.5 cm^3^ (Cassandri et al., 2021). Mice were irradiated at room temperature using an Elekta 6-MV photon linear accelerator. Three fractions of 2 Gy were delivered every other day, the first, third and fifth d, for a total dose of 6 Gy. A dose rate of 1.5 Gy/min was used with a source-to-surface distance (SSD) of 100 cm. Prior to irradiation, mice were anesthetized and were protected from off-target radiation by a 3 mm lead shield. Before tumor inoculation mice were randomly assigned to four experimental groups. Each group was composed of eight mice. One control group received 200 μl carrier solution by mouth (PO); PBI-05204 (20 mg/kg/5 Day/week (PO); one group received RT (3 fractions of 2 Gy delivered every other day to a total dose of 6 Gy); PBI-05204 (20 mg/kg/5 Day/week, (PO) coupled with RT (3 fractions of 2 Gy delivered every other day to a total dose of 6 Gy) (Colapietro et al., 2022). During treatment, mice with significant body weight loss approaching (10–15%) were euthanized early per protocol, by using Carbon Dioxide, following the AVMA Guidelines for the Euthanasia of Animals (AVMA, 2019). The effects on tumor growth because of different treatments were evaluated as follows: tumor volume was measured during and at the end of the experiment; tumor volume was assessed every 4 days with a Vernier caliper (length × width); the volume of the tumor was expressed in mm^3^ according to formula 4/3π r^3^, measuring tumor weight at the end of the experiment and defining tumor progression (TP), the doubling of the tumor volume.

### Statistical analysis and data analysis

Three independent experiments, each performed in triplicate, were carried out and the results were expressed as the mean ± SD. Assessment of normal distribution of data was confirmed by Shapiro–Wilk, D’Agostino and Pearson and Kolmogorov–Smirnov tests. Real-time PCR experiments were evaluated by one-way (ANOVA) with a Tukey’s post hoc test using 2^−ΔΔCT^ values for each sample. Flow cytometry data were analyzed by ANOVA with a Bonferroni post hoc test. All analyses were performed using the SAS System (SAS Institute Inc., Cary, NC, United States) and GraphPad Prism 6.1.

## Results

PBI-05204 Induces Concomitant Growth Arrest and Cell Death in RMS but not in MSCs Cell Lines *in vitro*.

**FIGURE 1:**
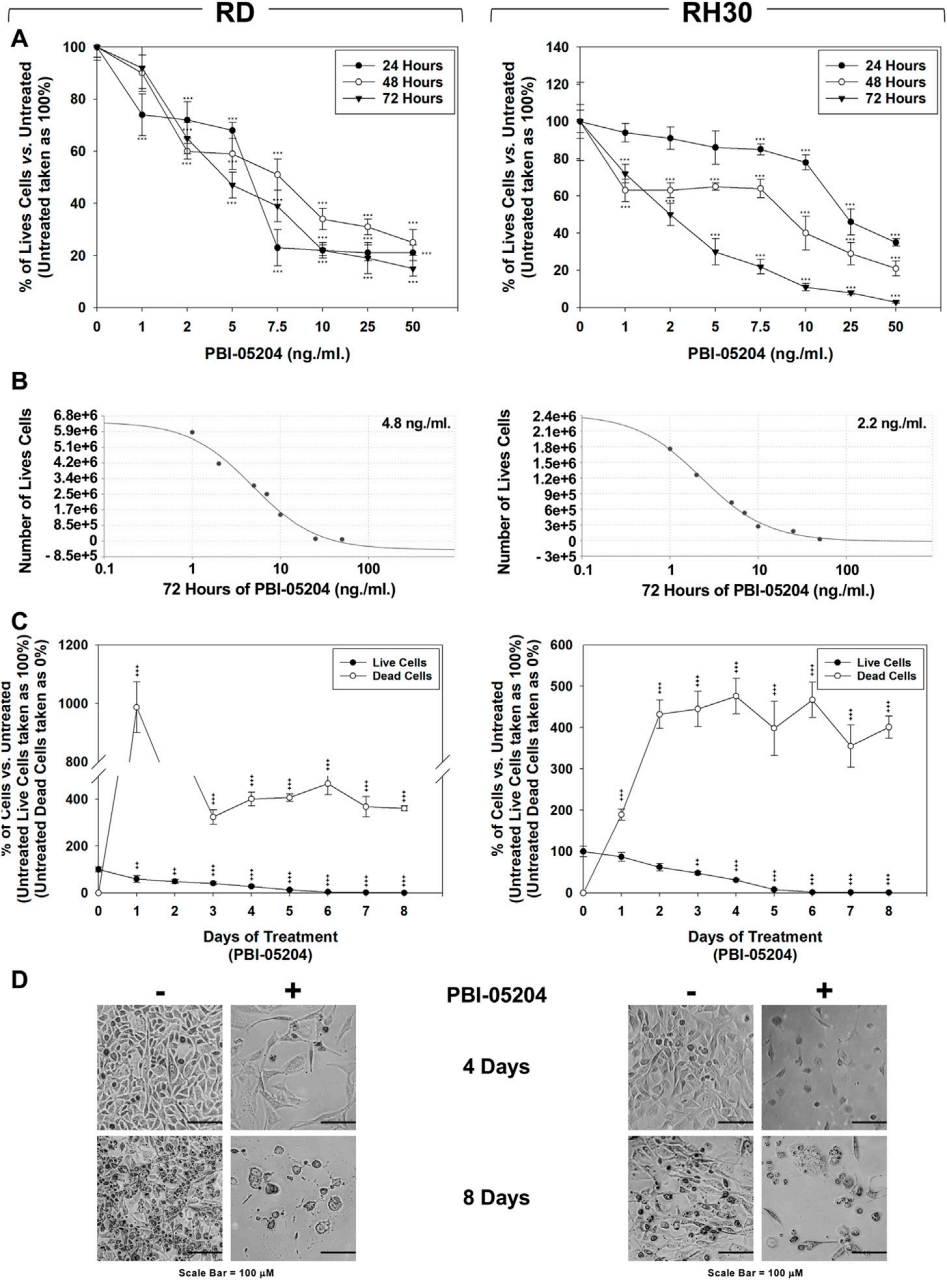
PBI-05204 induces concomitant cell death and growth arrest in FN-RMS and FP-RMS cells. RD (Left) and RH30 (Right) cell lines were treated for 24, 48 and 72 h with increasing concentrations (0, 1, 2, 5, 7.5, 10, 25, 50 ng/ml) of the drug. Surviving **(A)** cells were counted using Trypan blue dye exclusion test. **(B)** Concentration of PBI-05204 able to reduce by 50% the cell survival of RD (Left) and RH30 (Right) cell lines. **(C)** Effect of PBI-05204 IC_50_ on cell number of live and dead RD (Left) and RH30 (Right) cells. Surviving cells were counted using Trypan blue dye exclusion test. Results represent the mean values of three independent experiments ±SD. Statistical significance: *p ≤ 0.05, **p ≤ 0.01, ***p ≤ 0.001 vs. Untreated. **(D)** Cellular morphology of RD (Left) and RH30 (Right), untreated or treated with PBI-05204 (IC50) for 4 d was analyzed under light microscope at ×200 magnification.

Trypan blue dye exclusion test showed that increasing doses (0–50 ng/ml) of PBI-05204 treatment, performed for 24, 48 and 72 h, significantly reduced the number of live cells in a concentration-dependent manner, both in RD (Figure 1A, Left Panel) and RH30 cells (Figure 1A, Right Panels), with an average of 50% cell viability at a concentration of 4.8 ng/ml on RD (Figure 1B, Left Panel) and 2.2 ng/ml on RH30 cell line (Figure 1B, Right Panel). RMS cultures were treated with PBI-05204 (IC_50_) for 8 days. As shown in Figure 1C, 4 days of PBI-05204 (IC_50_) treatment reduced the number of living cells by 71.4 ± 4.7% in RD (Figure 1C, Left Panel, 4 d, Live Cells) and 78.3 ± 3.7% in RH30 cells (Figure 1C, Right Panel, 4 d, Live Cells). Prolonged drug exposure resulted in the total absence of viable cells (Figure 1C, RD, Left Panel, and RH30, Right Panel, Time 8 days, Live Cells). Concomitantly, the number of dead cells was progressively and persistently induced by the presence of PBI-05204 (Figure 1C, RD, Left Panel, and RH30, Right Panel. Notably, PBI-05204-treated adherent cells exhibited a substantial change in their morphology, with larger cellular bodies at 4 d post-exposure (Figure 1D, RD, Left Panel, and RH30, Right Panel, 4 d). Cell fragments and cells almost devoid of cytoplasm appeared after 8 days of continuous treatment (Figure 1D, RD, Left Panel, and RH30, Right Panel, 8 days). The IC_50_ of PBI-05204 was of 93.7 ng/ml for MSCs cells (Supplementary Data S1; Figure 1A), 19.5 and 42.5 times higher than RD and RH30, respectively. Notably, treating MSCs did not induce any statistically significant increase in dead cells (Supplementary Data S1; Figure 1B). Altogether, these data indicate that PBI-05204 induces concomitant growth arrest and cell death in RMS but not in the normal counterpart with FP-RMS being more sensitive to the drug than FN-RMS.

PBI-05204 Causes G_1_ Phase Cell Cycle Arrest of Both FN-RMS and FP-RMS and Senescence in FN-RMS.

**FIGURE 2:**
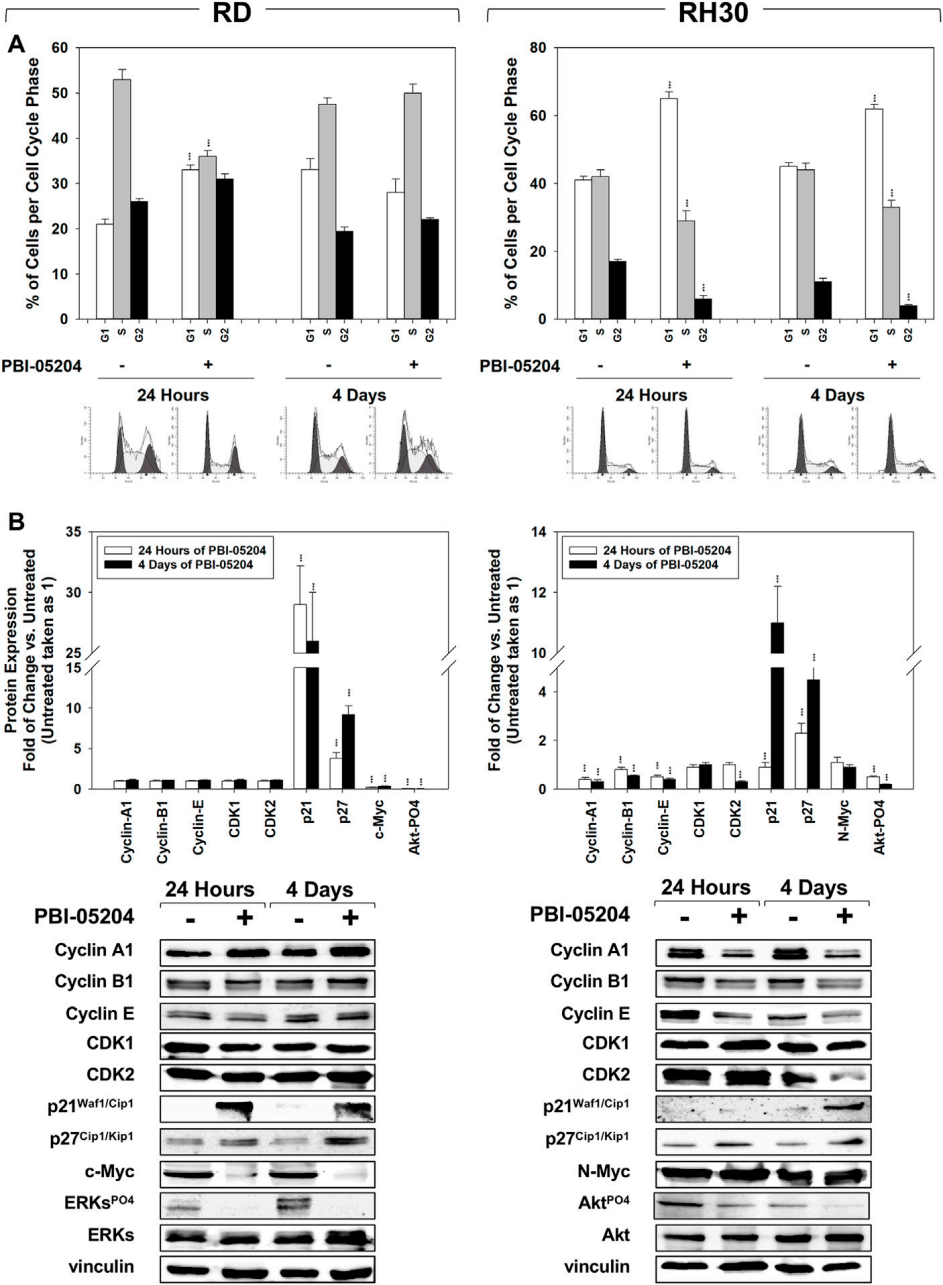
PBI-05204 affects cell cycle distribution. **(A)** FACS analysis performed on RD (Left) and RH30 (Right) untreated or treated for 4 with PBI-05204 (IC50). Data (Up) indicates the percentage of cells in each cell cycle phase representing the mean value of three independent experiments. (Down) Representative data of three independent experiments is shown. **(B)** Cell lysates from RD (Left) and RH30 (Right) cells treated for 24 h and 4 d with PBI-05204 (IC50) were analyzed by immunoblotting with specific antibodies for the indicated proteins; vinculin expression was used as a loading control. Histograms (Up) of densitometric analysis represent the mean values of three independent experiments ±SD. Statistical significance: **p ≤ 0.01, ***p ≤ 0.001 vs. Untreated cells. Representative data from three independent experiments is shown (Down).

**FIGURE 3:**
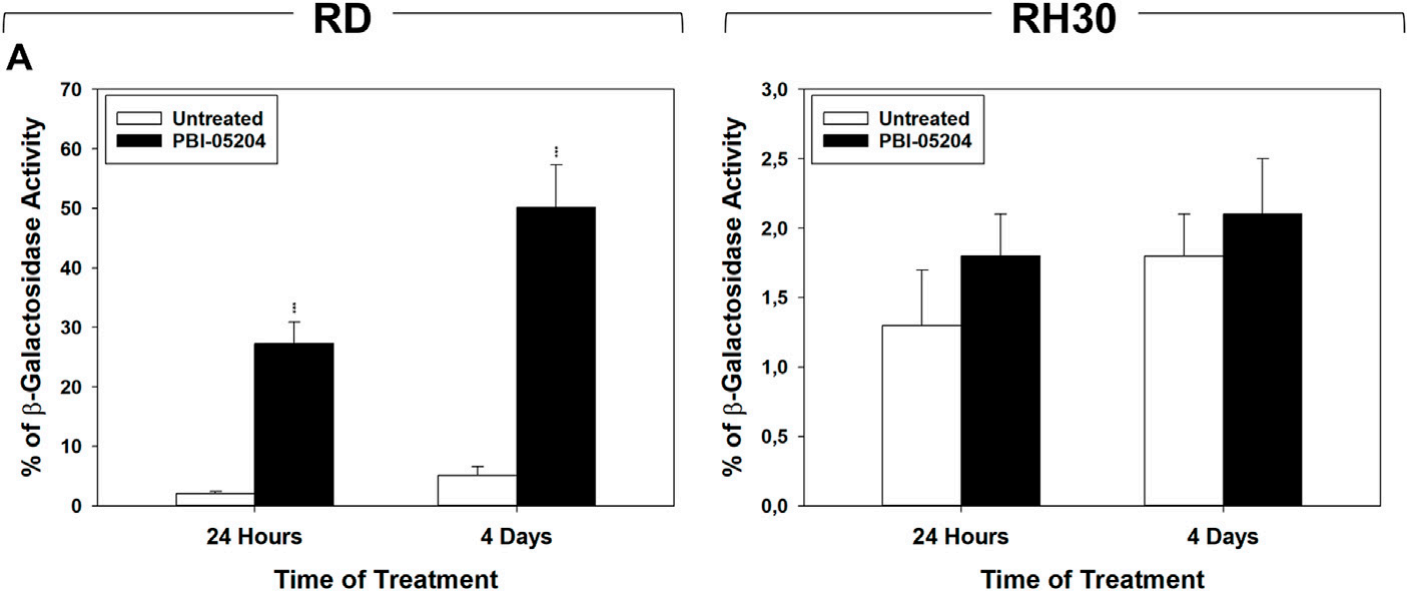
PBI-05204 induces senescence in FN-RMS but not in FP-RMS cells. **(A)** b-Galactosidase test performed on RD (Left) and RH30 (Right) cells untreated or treated for 24 h and 4 d with PBI-05204 (IC_50_). Data represent the mean value of three independent experiments ±SD. Statistical significance: **p ≤ 0.01, ***p ≤ 0.001 vs. Untreated cells.

**FIGURE 4:**
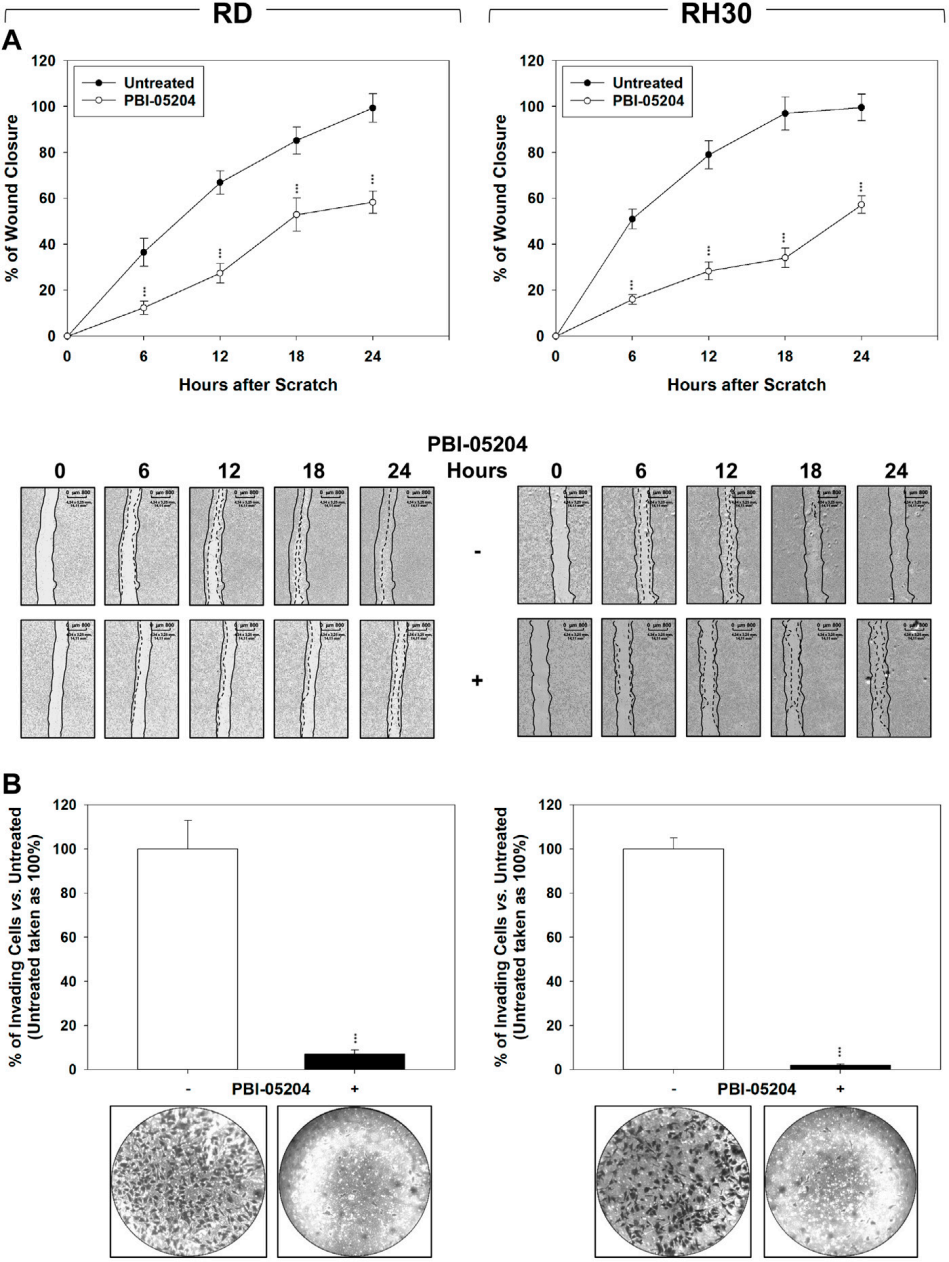
PBI-05204 impairs migration and invasion as determined by wound closure by FN-RMS and FP-RMS cells. **(A)** Wound healing experiments in RD (Left) and RH30 (Right) cell lines, treated or not with PBI-05204 IC_50_. The scratch and then PBI-05204 treatment was made at time 0 and maintained or not for 24 h. The dotted lines represent the edges of the wound. Photographs (Left Panel) were taken under light microscope (×10 magnification). The migration index was plotted in bar graphs as the % of wound area (Right Panel). Lines (Up) represent the mean values of three independent experiments ±SD. Statistical significance: **p ≤ 0.01, ***p ≤ 0.001 vs. Untreated cells. Representative data from three independent experiments is shown (Down). **(B)** Matrigel invasion assay using a Transwell system. Results represent the mean values of three independent experiments ±SD (Up). Statistical significance: *p ≤ 0.05, **p ≤ 0.01, ***p ≤ 0.001 vs. Untreated. Representative data from three independent experiments is shown (Down).

Cell cycle distribution analysis, performed by flow cytometry on RMS cells treated for 24 h and 4 days with PBI-05204 (IC_50_), showed that in RD cells this drug significantly, but transiently, arrested cells in the G_1_ phase. After 24 h of treatment with PBI-05204 the percentage of cells in G_1_ phase significantly increased (Figure 2A, RD 24 h, 33% ± 1.1 PBI-05204 vs. 21% ± 1.3 Untreated) by primarily reducing the number of cells in S phase (Figure 2A, RD 24 h, 36% ± 1.3 PBI-05204 vs. 53% ± 2.2 Untreated) while no statistically significant differences on cell cycle distribution were observed after 4 days of treatment (Figure 2A, RD 4 days). In RH30 cells, PBI-05204 induced a rapidly (24 h) and persistently (4 days) cell cycle arrest as indicated by the increase of cell number in the G_1_ phase (Figure 2A, RH30 24 h, G_1_ phase: 65% ± 2 PBI-05204 vs. 41% ± 1.2 Untreated and 4 days, G_1_ phase: 62% ± 1.4 PBI-05204 vs. 45% ± 1.1 Untreated) and the concomitant reduction in both the S phase (Figure 2A, RH30 24 h, S phase: 29% ± 3 PBI-05204 vs. 42% ± 2 Untreated and 4 days, S phase: 33% ± 2.1 PBI-05204 vs. 44% ± 2 Untreated) and the G_2_ phase (Figure 2A, RH30 24 h, G_2_ phase: 6% ± 1 PBI-05204 vs. 17% ± 0.6 Untreated and 4 d, G_2_ phase: 4% ± 0.4 PBI-05204 vs. 11% ± 1 Untreated). RD cells treated with PBI-05204 downregulated the expression of the cell cycle promoters c-Myc (Figure 2B, RD, c-Myc), but not of Cyclin A1 (Figure 2B, RD, Cyclin A1), Cyclin B1 (Figures 2B,D, Cyclin B1), Cyclin E (Figure 2B, RD, Cyclin E), CDK1 (Figure 2B, RD, CDK1) and CDK2 (Figure 2B, RH30, CDK2), and upregulated the expression of cell cycle inhibitors p21^Waf1/Cip1^ and p27^Cip1/Kip1^ (Figure 2B, RD, p21^Waf1/Cip1^ and p27^Cip1/Kip1^). On the other hand, consistent with G_1_ cell cycle arrest, RH30 cells treated with PBI-05204 showed the a downregulation of Cyclin A1 (Figure 2B, RH30, Cyclin A1), Cyclin B1 (Figure 2B, RH30, Cyclin B1), Cyclin E (Figure 2B, RH30, Cyclin E) and CDK2 expression levels (Figure 2B, RH30, CDK2), and an the upregulation of the expression of p21^Waf1/Cip1^ and p27^Cip1/Kip1^ cell cycle inhibitors (Figure 2B, RH30, p21^Waf1/Cip1^ and p27^Cip1/Kip1^), whilst no changes were observed in N-Myc and CDK1 protein levels (Figure 2B, RH30, N-Myc). Furthermore, PBI-05204 affected the phosphorylation/activation of ERKs in RD (Figure 2B, RD, ERKsPO4) and of Akt in RH30 (Figure 2B, RD, AktPO4, RH30). Due to PBI-05204-induced upregulation of p21^Waf1/Cip1^ and p27^Cip1/Kip1^, biomarkers of senescent cells (Flores et al., 2014; Kumari and Jat, 2021), the induction of senescence-associated β-galactosidase activity (SA-β-Gal), considered to be an important hallmark of cell senescence (Dimri et al., 1995), was evaluated on RMS cells after 24 h and 4 days of PBI-05204 (IC_50_) treatment. As shown in Figure 3, PBI-05204 increased SA-β-Gal 24 h later (Figure 3, RD 24 h, 26.3% ± 4,1 PBI-05204 vs. 2.3% ± 0,2 Untreated), up to 4 days (Figure 3, RD cell lines 4 d, 50.1% ± 8,3 PBI-05204 vs. 5.2% ± 0,6 Untreated) in RD whilst no statistically significant differences were obtained in RH30 (Figure 4, RH30 24 h and 3 days, PBI-05204 vs. Untreated). Thus, these data indicate that PBI-05204 induce the growth arrest of FP- and FN-RMS in two different ways: persistently perturbing the cell cycle distribution of RH30 cells and promoting the senescence in RD cells.

### PBI-05204 Inhibits the Ability of RMS to Migrate and Invade.

The ability of PBI-05204 to affect the migration/invasion ability of RMS cells was investigated. As shown in Figure 4A, PBI-05204 (IC_50_) reduced RMS cell migration as assessed by wound healing assays in which the same fields of confluent cells were pictured immediately after the scratch (time 0 h) and again after 6, 12, 18 and 24 h following drug pre-incubation. Particularly, 24 h after the scratch, PBI-05204 decreased the level of wound closure to 41.1% ± 4.9% for RD (Figure 4A, RD, 24 h Untreated vs. PBI-05204) and 42.4% ± 3.8% for RH30 of the control sample (Figure 4A, RH30, 24 h Untreated vs. PBI-05204). Furthermore, PBI-05204 inhibited the ability of both RD of 92.3% ± 2.1% and RH30 of 98.7% ± 1.1% to invade chambers coated with Matrigel (Figure 4B, RD and RH30 Untreated vs. PBI-05204). Altogether, these data indicate that PBI-05204 can also counteract the *in vitro* ability of RMS to migrate and invade.

### PBI-05204 sensitizes RMS cells to radiation affecting also the intrinsically radioresistant stem-like cell population

**FIGURE 5:**
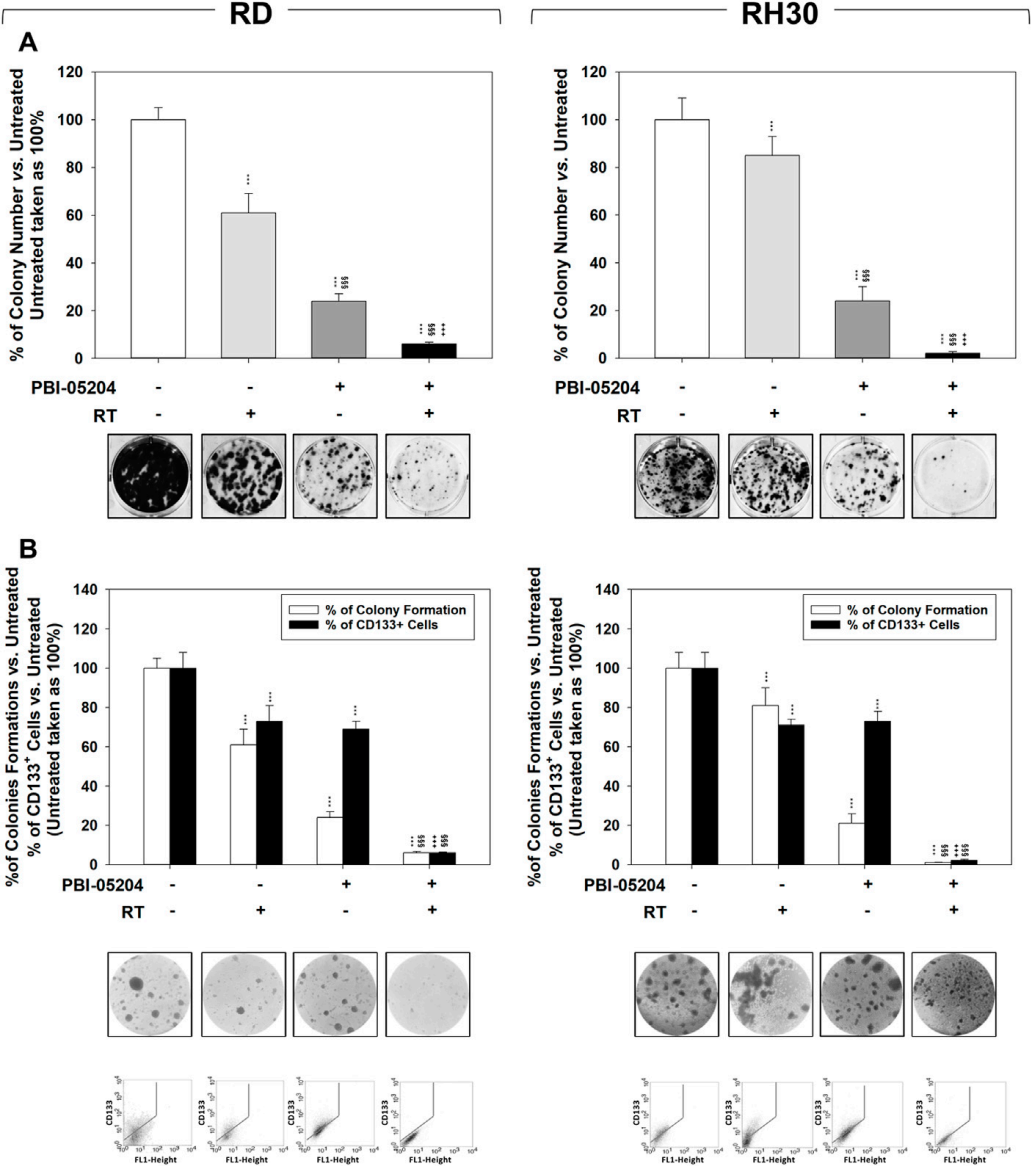
PBI-05204 radiosensitizes FN-RMS and FP-RMS cell lines and cancer-stem like derived cells. **(A)** Colony formation assay of RD (Left) and RH30 (Right) treated with PBI-05204 IC_50_, RT alone or with the combination. Three h after RT (4 Gy), cells were seeded at low concentrations for colony assays. Colony forming efficiency was calculated by crystal violet absorbance after 14 d of PBI-05204 treatment. **(B)** The formation of spheres enriched in stem-like cells and the expression of CD133 were assessed in RD (Left) and RH30 (Right) treated with PBI-05204 (IC50), RT alone or with the combination. Results represent the mean values ±SD of three independent experiments (Up). Representative data from three independent experiments is shown (Down). Statistical significance: *p ≤ 0.05, **p ≤ 0.01, ***p ≤ 0.001, vs. Untreated, §*p* ≤ 0.05, §§*p* ≤ 0.01, §§§*p* ≤ 0.001 vs. RT, +*p* ≤ 0.05, ++*p* ≤ 0.01, +++*p* ≤ 0.001 vs. PBI-05204.

**FIGURE 6:**
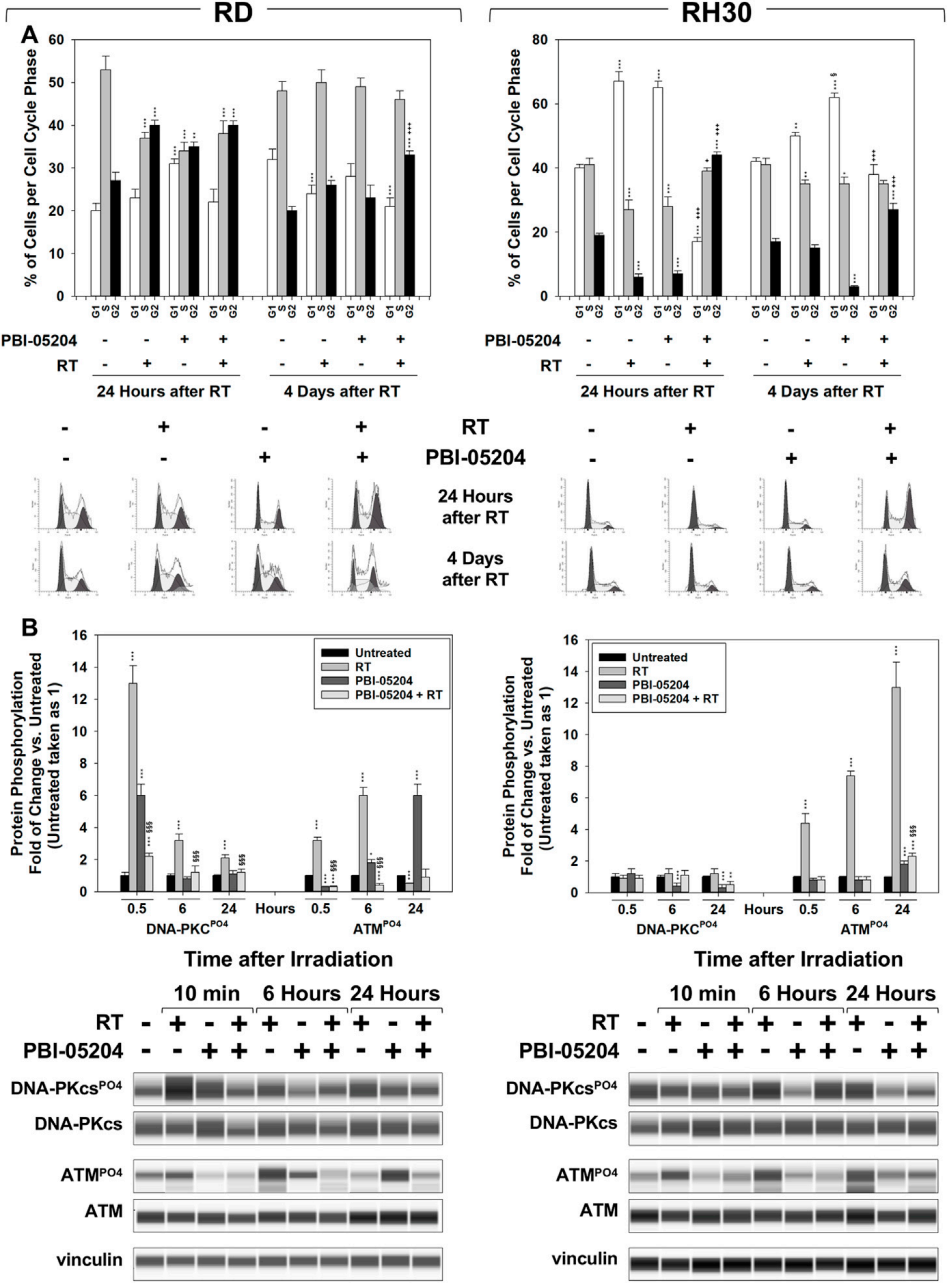
PBI-05204 promotes RT-induced accumulation of cancer cells in the G2 phase of the cell cycle and counteracts the ability of FN-RMS and FP-RMS to repair damaged DNA. **(A)** FACS analysis performed on RD (Left) and RH30 (Right) treated with PBI-05204 (IC_50_), RT alone or with the combination. Data (Up) show the percentage of cells in each cell cycle phase representing the mean value of three independent experiments. (Down) Representative data from three independent experiments is shown. **(B)** Cell lysates from RD (Left) and RH30 (Right) cells pre-treated for 24 h with PBI-05204 (IC50) and then irradiated with 4 Gy were collected 10 min, 6 and 24 h after RT. Cell lysates were analyzed by immunoblotting with specific antibodies for the indicated proteins; vinculin expression was used as a loading control. Histograms (Up) of densitometric analysis represent the mean values of three independent experiments ±SD. Statistical significance: **p ≤ 0.01, ***p ≤ 0.001 vs. Untreated cells, §*p* ≤ 0.05, §§*p* ≤ 0.01, §§§*p* ≤ 0.001 vs. RT, +*p* ≤ 0.05, ++*p* ≤ 0.01, +++*p* ≤ 0.001 vs. PBI-05204. A representative of three independent experiments is shown (Down).

The ability of PBI-05204 to sensitize cells to ionizing radiation was assessed through colony formation assay performed on RMS cells (Figure 5A) and RMS-derived CSC-like cells (Figure 5B) pre-treated for 24 h with PBI-05204 (IC_50_) and then irradiated with a dose of 4 Gy. The combination of RT and PBI-05204 improved the ability of this treatment to affect the clonogenic ability (Figure 5A) and to form tumor-spheres (Figure 5B) in both cell lines. The clonogenic ability was affected by RT alone of 39.1% ± 8% in RD (Figure 5A, RD, RT vs. Untreated) and 18% ± 8% in RH30 (Figure 5A, RH30, RT vs. Untreated), by PBI-05204 alone of 76% ± 3% in RD (Figure 5A, RD, PBI-05204 vs. Untreated) and 79% ± 5% in RH30 (Figure 5A, RH30, PBI-05204 vs. Untreated), and by RT combined with PBI-05204 by 94% ± 0.8% in RD (Figure 5A, RD, PBI-05204 + RT vs. Untreated) and 98% ± 0.4% in RH30 (Figure 5A, RH30, PBI-05204 + RT vs. Untreated). Drug treatment in combination with RT significantly reduced the rhabdosphere formation by 94.3% ± 0.6% in RD (Figure 5A, RD, PBI-05204 + RT vs. Untreated) and 98.2% ± 0.4% in RH30 (Figure 5A, RH30, PBI-05204 + RT vs. Untreated), significantly improving the efficiency of RT alone by 92.8% ± 2.9% in RD (Figure 5A, RD, PBI-05204 + RT vs. RT) and 97.5% ± 3.2% in RH30 (Figure 5A, RH30, PBI-05204 + RT vs. RT) and PBI-05204 alone by 77.3% ± 3.1% in RD (Figure 5A, RD, PBI-05204 + RT vs. PBI-05204) and 90.8% ± 2.4% in RH30 (Figure 6A, RH30, PBI-05204 + RT vs. PBI-05204). Notably, 24 h of PBI-05204 pre-treatment alone affected the formation of tumorspheres in RD by 90.5% ± 3.8% (Figure 5B, RD, Upper Panel, PBI-05204 vs. Untreated) and in RH30 by 98.8% ± 2.6% (Figure 5B, RH30, Upper Panel, PBI-05204 vs. Untreated) and, parallelly, the number of the CD133^+^ RMS cells (Figure 5B, RD and RH30, Lower Panel, PBI-05204 vs. Untreated). Thus, PBI-05204 can radiosensitize RMS independently from FP status, also targeting the CSC subpopulation.

### PBI-05204 promotes RT-induced G_2_ phase cell cycle arrest and impairs DNA double-strand break repair in RMS

The effects of PBI-05204 on cell cycle distribution and DNA repair were investigated. Cell cycle distribution analysis was performed by flow cytometry on RMS cells pre-treated for 24 h with PBI-05204 (IC_50_) and then irradiated with 4 Gy, 24 h, and 4 days after RT. As shown in Figure 6A, on RD cells, PBI-05204 pre-treatment increased the percentage of cells arrested in the G_2_ cell cycle phase by RT, 4 days after irradiation (Figure 6A, RD, 4 d, 30.7% ± 1.3% PBI-05204 + RT vs. 24.6% ± 2.3% RT). On RH30, PBI-05204 pre-treatment induced an early, 24 h (Figure 6A, RH30, 24 h, 43.3% ± 0.6% PBI-05204 + RT vs. 7.6% ± 1.2% RT), and stable G_2_ cell cycle phase arrest following 4 d (Figure 6A, RH30, 4 d, 28.3% ± 1.6% PBI-05204 + RT vs. 17.4% ± 1.1% RT) of drug exposure. The phosphorylation/activation status of DNA-PKcs and ATM, respectively upstream of Non-Homologous End-Joining (NHEJ) and Homologous Recombination (HR) DSB repair pathways, were also investigated. Pre-treating cells with PBI-05204 counteracted the RT-induced phosphorylation/activation of DNA-PKcs (Figure 6B, DNA-PKcsPO4 PBI-05204 + RT vs. RT), and ATM (Figure 6B, ATMPO4 PBI-05204 + RT vs. RT) in both RD and RH30 cells. Thus, the data suggest that PBI-05204 can sensitize FN-RMS and FP-RMS to RT by promoting the accumulation of cells in the G_2_, the most radiosensitive phase of the cell cycle (Pawlik and Keyomarsi, 2004), thereby impairing the ability of FN-RMS and FP-RMS to repair RT-induced DSBs.

### PBI-05204 radiosensitizes RMS cells *in vivo*

**FIGURE 7:**
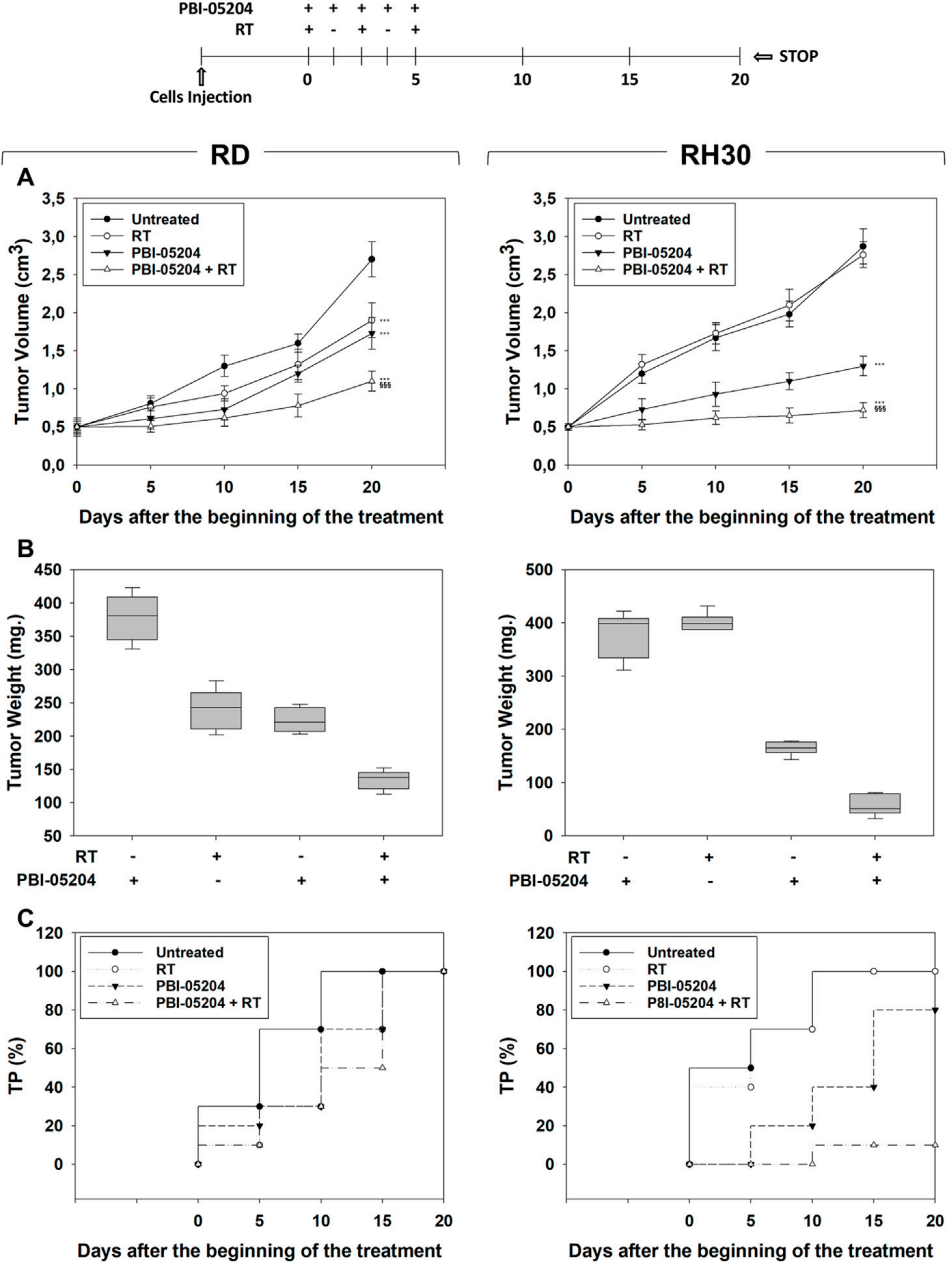
Effect of PBI-05204 combined or not with irradiation on *in vivo* tumor growth. The diagram above all the figures indicates the experiment procedure. **(A)** Growth curve of tumor volumes from xenografted RD and RH30 cell lines, untreated, PBI-05204-treated, irradiated (RT), PBI-05204-pre-treated and irradiated (RT + PBI-05204). Tumor volumes were evaluated as described in methods and represent the mean ± SEM of eight mice per group. The graphs show the sequential treatments of xenografted mice started when tumors reached an initial volume of approximately 0.5 cm^3^. Results represent the mean values ±SD. Statistical significance: **p ≤ 0.01, ***p ≤ 0.001 vs. Untreated mice; $$$ *p* ≤ 0.001 vs. RT-treated mice; ###*p* ≤ 0.001 vs. PBI-05204-treated mice. **(B)** Tumor weights from mice injected with RD (Left) and RH30 (Right) and treated with PBI-05204 and RT alone or in combination. **(C)** Kaplan–Meier estimates for rates of progression for untreated, PBI-05204, RT, or PBI-05204 + RT combination in RMS-derived tumors.

*In vivo* experiments were then performed by subcutaneously injecting (SC) RMS cells in nude mice. When the tumor volume reached 0.5 cm^3^ (T0), mice received PBI-05204 (20 mg/kg) (Colapietro et al., 2022) or vehicle (PBS) by mouth once daily for five consecutive d (see Methods) and then were irradiated, or not, with 2 Gy on the 1st, 3rd, and fifth d with PBI-05204 given 1 h before RT. Tumor volumes were measured every 5 days for a period of 20 days after the start of treatment. Compared to single treatments, combining RT and PBI-05204 significantly improved the therapeutic efficiency of RT resulting in 60.4% ± 6.3% volume reduction in RD (Figure 7A, RD, PBI-05204 + RT vs. RT) and 83.3% ± 6.2% in RH30 (Figure 7A, RH30, PBI-05204 + RT vs. RT) xenografts compared to RT alone, and of 37.5% ± 5.7% in RD (Figure 7A, RD, PBI-05204 + RT vs. PBI-05204) and 54.4% ± 8.1% in RH30 (Figure 7A, RH30, PBI-05204 + RT vs. PBI-05204) xenografts compared to PBI-05204 alone. Notably, PBI-05204 alone significantly affected the *in vivo* tumor growth of RD by 44% ± 3.9% (Figure 7A, RD, PBI-05204 vs. Untreated) and of RH30 by 52.1% ± 7.1% (Figure 7A, RH30, PBI-05204 vs. Untreated). Accordingly, tumor weights of xenografts from mice co-treated with PBI-05204 and RT decreased significantly compared to those of untreated mice and single treatments (Figure 7B, RH30, PBI-05204 vs. Untreated). In both RD and RH30 xenografted mice PBI-05204 and RT co-treatment slowed the tumor progression (TP) compared to PBI-05204 (Figure 7C, RD and RH30, PBI-05204 + RT vs. PBI-05204) or RT (Figure 7C, RD and RH30, PBI-05204 + RT vs. RT) alone. Taken together, these findings highlight the ability of PBI-05204 to radiosensitize both FN-RMS and FP-RMS with a greater effect on FP-RMS.\

## Discussion

Herein, the therapeutic potential of PBI-05204, a supercritical CO_2_ extract of *N. oleander* (Colapietro et al., 2022) already clinically successfully tested for its tolerability and safety in cancer patients (Hong et al., 2014; Roth et al., 2020), has been investigated in RMS cells, as a single agent and in combination with RT.

As previously demonstrated with *in vitro* and *in vivo* models of pancreatic cancer (Pan et al., 2015) and glioblastoma (Colapietro et al., 2020, 2022), PBI-05204 counteracted, *in vitro*, the aberrant proliferation of RMS, by concomitantly inducing cytostatic and cytotoxic effects in a concentration dependent manner. Notably, the half maximal inhibitory concentration (IC_50_) able to induce growth arrest was 4.8 ng/ml in FN- and 2.2 ng/ml in FP-RMS, suggesting a higher sensitivity to PBI-20054 of the most aggressive RMS subtype. Furthermore, prolonged treatment induced the death of all cells, suggesting that the initial cytostatic effect induced by PBI-05204 could be a failed attempt by cells to resist the cytotoxic action of the drug.

Treatment of FP-RMS resulted rapidly (24 h) and persistently (4 days) in an arrest in the G_2_/M phase of the cell cycle, while FN-RMS was transiently restrained in the G_1_/S phase, with a different molecular pattern between RMS subtypes.

In FP-RMS, PBI-05204 downregulated the expression of cyclin A1 and cyclin B1, promoters of G_2_/M transition (Jackman and Pines, 1997), and upregulated the expression of p21^Waf1/Cip1^ and p27^Cip1/Kip1^, cell cycle inhibitors globally acting in any phase of the cell cycle (Abukhdeir and Park, 2008). According to previously reported evidence (Hong et al., 2014), PBI-05204 inhibited the PI3-kinase/AKT protein kinase pathway. PI3-kinase/AKT signaling, known to be aberrantly activated and sustained in the transformed phenotype of FP-RMS (Sorensen et al., 2002; Davicioni et al., 2009; Rudzinski et al., 2015, 2017; Skapek et al., 2019), has multiple roles in regulation of cell cycle progression (Liang and Slingerland, 2003), including the G_2_/M phase (Shtivelman et al., 2002) in part by phosphorylating/inhibiting p21^Waf1/Cip1^ and p27^Cip1/Kip1^ (Chen et al., 2019). Furthermore, targeted inhibition of the PI3K/AKT pathway has been shown to enhance cell death in FP-RMS (Liu et al., 2009; Crose and Linardic, 2011). Thus, we believe that the cytostatic and cytotoxic effects concomitantly induced by PBI-05204 could be related to PI3K/AKT pathway inhibition. Notably, we noticed that PBI-05204 also downregulated the expression of cyclin E/CDK2 complex, known to be a G_1_ cell cycle promoter (Hinds et al., 1992). However, it has been shown that cyclin E/CDK2 complex can also promote non-cell cycle-related (Hydbring et al., 2016; Liu et al., 2019) pro-oncogenic functions (Hwang and Clurman, 2005; Pang et al., 2020), including in FP-RMS (Takahashi et al., 2004; Tamura et al., 2022). Thus, although we cannot exclude a possible role in growth arrest, we believe that PBI-05204-induced cyclin E/CDK2 downregulation is relevant in terms of affecting the relative aggressiveness FP-RMS.

In contrast to FP-RMS, PBI-05204-treatment did not modulate the expression of cyclins and/or CDKs in FN-RMS but downregulated the expression of the oncoprotein c-Myc. Similarly, PBI-05204 upregulated the expression of p21^Waf1/Cip1^ and p27^Cip1/Kip1^. We have previously shown that overexpression of p21^Waf1/Cip1^ (Ciccarelli et al., 2005) or downregulation of c-Myc itself (Gravina et al., 2016) can induce growth arrest in FN-RMS. Considering that PBI-05204 induces the dephosphorylation/inhibition of ERKs, known to be aberrantly activated in FN-RMS (Sorensen et al., 2002; Davicioni et al., 2009; Rudzinski et al., 2015, 2017; Skapek et al., 2019), upregulates p21^Waf1/Cip1^ (Ciccarelli et al., 2005) and downregulates c-Myc (Marampon et al., 2006) expression, the molecular modulations induced by PBI-05204 in FN-RMS may occur through disruption of the MEKs/ERKs signaling and consequent c-Myc-downregulation-mediated p21Waf1/Cip1 expression (Gartel et al., 2001) and p27^Cip1/Kip1^ phosphorylation/activation (García-Gutiérrez et al., 2019). Notably, according to the proposed roles of p21^Waf1/Cip1^ and p27^Cip1/Kip1^ as key master regulators of cellular senescence (Flores et al., 2014), a potentially reversible type of cell cycle arrest (Flores et al., 2014; Kumari and Jat, 2021), it was demonstrated that PBI-05204 increased the expression levels of SA-β-Gal, a marker of cell senescence (Dimri et al., 1995), in FN-RMS but not in FP-RMS. It has been recently shown that senescence is not always definitive and that arrested cancer cells can use senescence as an adaptive pathway to restart proliferation (Guillon et al., 2019). Thus, we speculate that the lower sensitivity of FN-RMS to PBI-05204 could be related to an ability of this RMS subtype to activate a program of senescence-mediated cellular protection in which an inability to be activated in FP-RMS makes the latter more sensitive to the drug.

The potential therapeutic efficiency of PBI-05204 is also suggested by its ability to counteract the growth of the cancer stem cell (CSC) subpopulation, as suggested by the assays based on the formation of CSCs-like enriched tumorspheres and the reduction in the expression of the stem cell marker CD133. The subpopulation of CSCs has been shown to drive tumor initiation, resistance to therapies and be responsible for local relapses and distant metastases (Phi et al., 2018; Arnold et al., 2020). Notably, according to the role of CSCs in promoting cancer metastases and the ability of PBI-05204 to counteract RMS stemness, the treatment reduced the ability of FN- and FP-RMS to migrate and invade. It has been recently shown that MEKs/ERKs signaling in FN-RMS and PI3K/AKTs signaling in FP-RMS sustain CSCs and a pro-metastatic phenotype (Ciccarelli et al., 2016; Manzella et al., 2020; Ramadan et al., 2020; Skrzypek et al., 2020). Thus, inhibition of these pathways by PBI-05204 could be explained at least in part by its ability to counteract stemness and the migratory behavior/metastatic potential of RMS cancer cells.

Since monotherapy frequently fails to adequately control tumor proliferation and RT has been shown to play a critical role in treating RMSs patients, the ability of PBI-05204 to increase the therapeutic efficacy of RT was investigated. PBI-05204 radiosensitized both FN- and FP-RMS, as indicated by the impaired clonogenic survival of PBI-05204-pre-treated non-CSCs and CSCs RMS cells. Since CSCs have been shown to contribute significantly to radiation resistance, and PBI-05204 has recently been shown to radiosensitize CSCs of GBM (Colapietro et al., 2022), it is suggested that this botanical drug is a strong candidate for use as a radiosensitizer in the treatment of RD. The molecular mechanisms involved in radioresistance are not fully understood (Arnold et al., 2020), even though DNA repair remains the mechanism primarily responsible for resistance of cancer cells to radiation (Schulz et al., 2019). A tumor cell’s ability to effectively repair DNA damage can enable it to: 1) resume proliferation, abandoning the redistribution of the cell cycle induced by radiation; 2) effectively repopulate the portion of cancer cells killed by radiation; 3) counteract the radiosensitizing phenomenon of reoxygenation; and, 4) acquire a more aggressive phenotype capable of increasing intratumoral heterogeneity, reducing the immunostimulating potential of RT (Boustani et al., 2019). Homologous recombination (HR), mediated by ATM signaling, and NHEJ, mediated by DNA-PKcs signaling, are the two major pathways for repair of DSBs, (Toulany, 2019). PBI-05204 efficiently, stably, and persistently counteracted the phosphorylation/activation of ATM on both FN- and FP-RMS. ATM contributes to radioresistance of several cancer cell types (Tribius et al., 2001; ZHOU et al., 2013; Bernardino-Sgherri et al., 2021; Cao et al., 2021; Zhang et al., 2022), resulting in an important pathway for DSB repair (Helleday, 2010), as also confirmed by García et al., 2022. Recently, a Phase I clinical trial investigating the radiosensitizing efficiency of M3541, a selective inhibitor of ATM, has closed unsuccessfully (Waqar et al., 2022), while another, assessing the safety and tolerability of the ATM inhibitor AZD1390 in combination to RT in patients with brain cancer is ongoing (NCT03423628). It has been largely shown that cancer cells can activate alternative survival pathways as a major mechanism of drug resistance and this also involves resistance to DNA repair inhibitors (Baxter et al., 2022). In this context, having available a drug capable of inhibiting the main pathways responsible for rhabdomyosarcomagenesis and DNA repair is certainly an advantage. That PBI-05204 can counteract in a stable and persistent way the state of phosphorylation/activation of DNA-PKcs, and thereby abolish repair of DSBs in the most aggressive and radioresistant subtype of RMS, the FP-RMS, represents a further point in favor of the use of PBI-05204 as a radiosensitizing agent. Further research is required to understand why inhibition of DNA-PKcs is transient in FN-RMS and whether this can be interpreted as a potential attempt to activate a resistance mechanism. However, PBI-05204 efficiently counteracted the ability of RMS to escape from RT-induced G2/M growth arrest on both FN- and FP-RMS, predisposing cancer cells to a higher sensitivity to subsequent fractions of irradiation (Toulany, 2019).

Finally, PBI-05204 radiosensitized FN- and FP-RMS in *in vivo* xenograft models of RMS as indicated by the ability of RT to counteract tumor growth and progression more efficiently in PBI-05204 pre-treated mice. The dose of PBI-05204 (20 mg/kg) (Colapietro et al., 2022) herein used in *in vivo* experiments is effectively higher than used in the phase I clinical trial (Hong et al., 2014). This is explained by the fact that mice lack expression of the Na, K-ATPase alpha3 subunit, which is important for efficient uptake of oleandrin, the major bioactive component of PBI-05204 (Yang et al., 2009), which leads to a relatively low absorption. However, no differences were noticed between the blood concentration of oleandrin in treated mice (Pan et al., 2015) and patients treated with the highest dose of PBI-05204 (Hong et al., 2014). Additional research requires that lower doses of PBI-05204 will need to be evaluated.

RT remains a pivotal treatment in the management of RMS even though a significant number of patients experience local and/or distant recurrences. The use of hypofractionated schedules has not improved the therapeutic efficiency of RT. Therefore, there is an urgent need to identify new radiosensitizing strategies. The data reported herein suggests that PBI-05204 could be an important new radiosensitizer with strong activity toward FP-RMS, the most aggressive type of RMS.

## Data Availability

The original contributions presented in the study are included in the article/Supplementary Materials, further inquiries can be directed to the corresponding author.

## Supplementary material

SUPPLEMENTAL FIGURE S1 Multipotent mesenchymal stromal cells (MSCs) are slightly sensitive to PBI-05204. Dose of PBI-05204 able to reduce by 50% the cell survival of MSCs cells. **(B)** Effect of PBI-05204 IC50 on cell number of dead MSCs cells. Surviving cells were counted using Trypan blue dye exclusion test. Results represent the mean values of three independent experiments ± SD. Statistical significance: *p ≤ 0.05, **p ≤ 0.01, ***p ≤ 0.001, Live Cells, Untreated vs. PBI-05204.
