# Supplementary figures and images for: The botanical drug PBI-05204, a supercritical CO2 extract of Nerium oleander, sensitizes alveolar and embryonal rhabdomyosarcoma to radiotherapy in vitro and in vivo
Source: Front Pharmacol. 2022 Dec 1;13:1071176. doi: 10.3389/fphar.2022.1071176 (PMC9751381; doi:10.3389/fphar.2022.1071176)

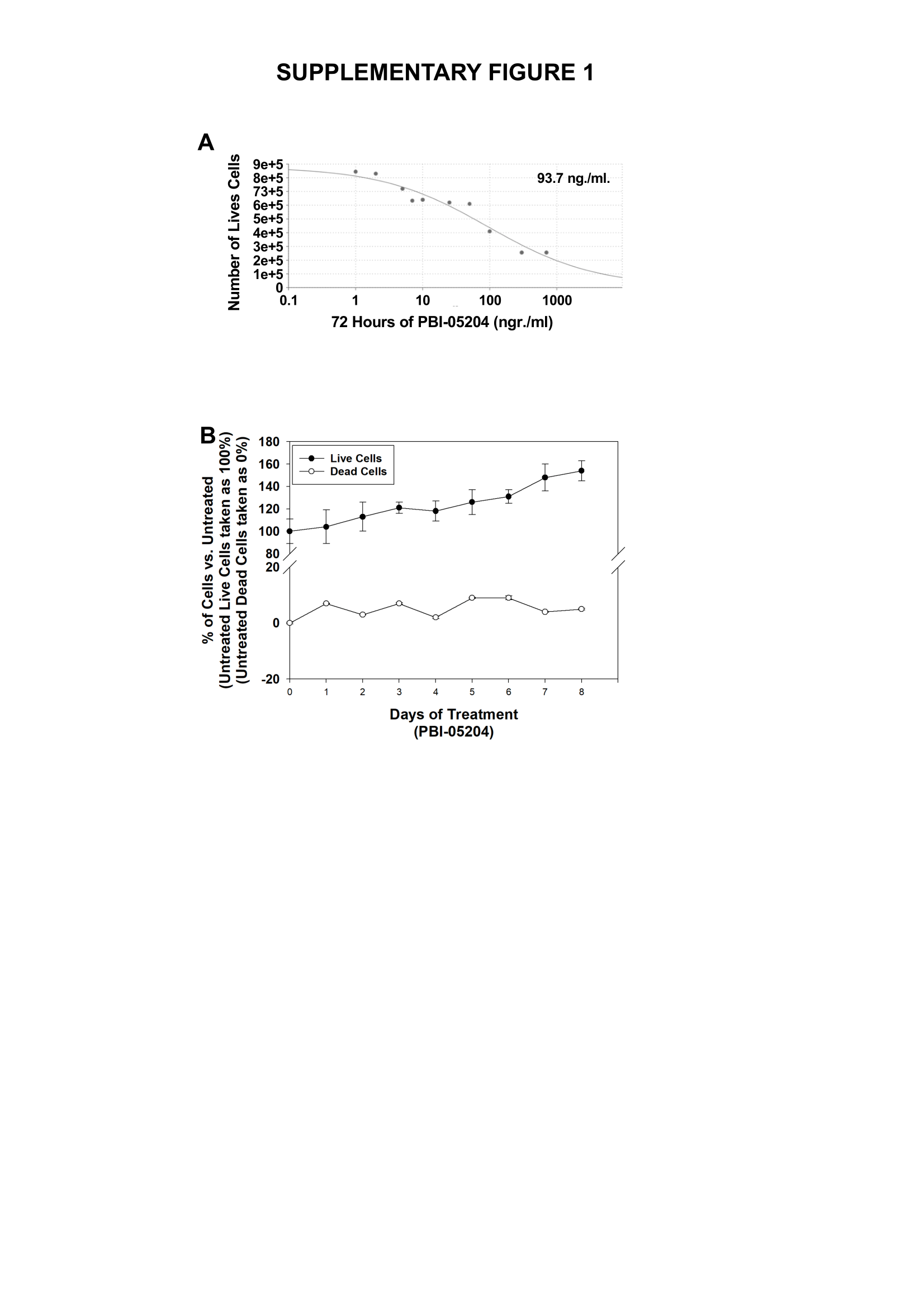

Supplement: Supplementary file 1 [file Image1.tiff]
